# Longitudinal follow-up of metformin treatment in Fragile X Syndrome

**DOI:** 10.3389/fpsyg.2024.1305597

**Published:** 2024-06-13

**Authors:** Panhaneath Seng, Federica Alice Maria Montanaro, Hazel Maridith Barlahan Biag, Maria Jimena Salcedo-Arellano, Kyoungmi Kim, Matthew Dominic Ponzini, Flora Tassone, Andrea Schneider, Leonard Abbeduto, Angela John Thurman, David Hessl, Francois V. Bolduc, Sebastien Jacquemont, Sarah Lippé, Randi J. Hagerman

**Affiliations:** ^1^MIND Institute, University of California Davis Health System, Sacramento, CA, United States; ^2^Child and Adolescent Neuropsychiatry Unit, Bambino Gesù Children’s Hospital, IRCCS, Rome, Italy; ^3^Department of Education, Psychology, Communication, University of Bari Aldo Moro, Bari, Italy; ^4^Department of Pediatrics, University of California Davis School of Medicine, Sacramento, CA, United States; ^5^Department of Public Health Sciences, University of California Davis School of Medicine, Sacramento, CA, United States; ^6^Integrative Genetics and Genomics Graduate Group, University of California Davis, Davis, CA, United States; ^7^Department of Biochemistry and Molecular Medicine, University of California Davis School of Medicine, Sacramento, CA, United States; ^8^Department of Psychiatry and Behavioral Sciences, University of California Davis School of Medicine, Sacramento, CA, United States; ^9^Department of Pediatrics, Department of Medical Genetics, Women and Children Health Research Institute, University of Alberta, Edmonton, AB, Canada; ^10^CHU Sainte-Justine Research Center, Université de Montréal, Montreal, QC, Canada; ^11^Department of Pediatrics, University of Montreal, Montreal, QC, Canada; ^12^Department of Psychology, Université de Montréal, Montreal, QC, Canada

**Keywords:** metformin, fragile X syndrome, treatment, IQ, adaptive behavior, longitudinal follow-up

## Abstract

**Introduction:**

Metformin has been used as a targeted treatment to potentially improve cognition and slow the typical IQ decline that occurs during development among individuals with fragile X syndrome (FXS). In this follow-up study, we are following the trajectory of IQ and adaptive behavior changes over 1 to 3 years in individuals with FXS who are clinically treated with metformin in an open label trial.

**Method:**

Individuals with FXS ages 6 to 25 years (mean 13.15 ± 5.50) and nonverbal IQ mean 57.69 (±15.46) were treated for 1–3 years (1.88 ± 0.63). They all had a baseline IQ test using the Leiter-III non-verbal cognitive assessment and the Vineland-III adaptive behavior assessment before the start of metformin. Repeat Leiter-III and Vineland-III were completed after at least 1 year of metformin (500–1,000 mg/dose given twice a day).

**Result:**

There were no significant changes in non-verbal IQ or in the adaptive behavior measurements at FDR < 0.05. The findings thus far indicate that both IQ and adaptive behavior are stable over time, and we did not see a significant decline in either measure.

**Conclusion:**

Overall, the small sample size and short follow-up duration limit the interpretation of the effects of metformin on cognitive development and adaptive functioning. There is individual variability but overall for the group there was no significant decline in IQ or adaptive behavior.

## Introduction

Fragile X Syndrome (FXS) (OMIM #300624) is the most common inherited neurodevelopmental cause of intellectual disability (ID) and autism spectrum disorder (ASD) due to a single gene. It is an X-linked genetic disorder, whose prevalence is approximately 1 in 4,000 males and 1 in 6,000 females (Coffee et al., 2009). It is due to the expansion of a cytosine—guanine—guanine (CGG) trinucleotide sequence on the 5’untranslated promoter region of the Fragile X Messenger Ribonucleoprotein 1 (*FMR1*) gene located at Xq27.3 (Hagerman et al., 2017). The expansion to more than 200 CGG repeats is the full mutation (FM) leading to hypermethylation of the *FMR1* gene and transcriptional silencing of the protein from *FMR1* (FMRP), which is essential for synaptic development, neurologic and cognitive function (Richter and Zhao, 2021); thus, its reduction/absence is associated with a wide range of physical features and cognitive-behavioral phenotypes (Palumbo et al., 2023). Physical features may include large ears, long face, flat feet and various medical problems (McLennan et al., 2011). However, what most impacts the quality of life and long-term prognosis of people with FXS includes ID, adaptive functioning deficits and behavioral problems.

In general, the clinical presentation and degree of disability of FXS depends on the amount of FMRP that is produced, which in part depends on *FMR1* methylation (Usdin et al., 2014). Males with FM are generally more affected, as they typically do not produce FMRP, while in females with FM, FMRP can range from close to normal to significantly reduced. Furthermore, females with FXS present a milder phenotype due to the presence of the unaffected X chromosome. Consequently, while more than 90% of males exhibit ID (mostly moderate), only the 30–50% of females show IQ scores less than 70 (Salcedo-Arellano et al., 2020). Together with ID, most males with FXS experience language delay, attention-deficit/hyperactivity disorder (ADHD) and ASD (Hagerman et al., 2009). Adaptive functioning deficits have been reported too, with relatively more preserved abilities in Daily Living and Motor domains (i.e., Fisch et al., 2007) and weaknesses in Socialization and Communication abilities (Hahn et al., 2015). Research has shown that FXS is associated with IQ decline over time (Lachiewicz et al., 1987; Dykens et al., 1989; Wright-Talamante et al., 1996), which is related to slower development particularly with abstract reasoning compared to typical peers so that IQ declines with age but abilities are not lost, although the cognitive deficits have a dramatic effect on the economic burden for families and society (Opitz, 1984; Hagerman et al., 1985).

Lachiewicz et al. (1987) carried out the first longitudinal study with male individuals with FXS, who were tested twice over the age span from 5 to 19 years, highlighting a significant deterioration from the first to the second assessment. Again, Dykens et al. (1989) conducted a longitudinal study with males with FXS, depicting a pattern of IQ trajectory of relative stability and steady growth until the ages of approximately 10 to 15, after which IQ declines (Dykens et al., 1989). In the same year, Hagerman et al. (1989) observed a cognitive decline in males with FXS aged between 8 and 12. These results were replicated by Hodapp et al. (1990) in a larger number of males with FXS under the age of 21, suggesting a widespread decline between 11 and 15 years of age. Four years later, Fisch et al. (1994) conducted the first preliminary longitudinal study about IQ decline in females with FXS, showing that deterioration occurs in both sexes, not only in males. This data was confirmed by Wright-Talamante et al. (1996), who performed a controlled retrospective study of longitudinal changes in IQ scores in females and males with FXS, pointing out that IQ declined in both sexes, with no significant differences between males and females. Later, Hall et al. (2008) implemented the first study in which IQ change assessed over two time points of children with FXS was compared to the one of their unaffected siblings, controlling the effects of age, sex, and FMRP levels. They observed a strong correlation between FMRP amount and ID severity in both the evaluations even though, when including age, sex, and time of assessment as covariates, FMRP accounted for only 5% of the variance. On the other hand, they found a high association between FMRP levels and sex. This study is important because for the first time FMRP levels have been correlated with ID severity in males and females with FXS.

Later, partially in contrast to the studies just described, Frolli et al. (2015) longitudinally evaluated the cognitive and adaptive functioning of 26 adolescents with FXS (with Wechsler Intelligence Scales-Revised—WISC-R and the Vineland Adaptive Behavior Scales—VABS), finding that Non-verbal IQ significantly decreased, whereas Verbal IQ improved, and Full-Scale IQ remained unchanged over time. In any case, regardless of the differences between studies, there is consistent evidence of IQ decline with aging over time in people with FXS. Likewise, research on the developmental trajectories of adaptive behavior in FXS, has shown declines over the time, with a trend that mirrors the IQ deterioration during adolescence (Freund et al., 1995; Fisch et al., 1999, 2002; Klaiman et al., 2014). Interestingly, Frolli et al. (2015) in the same study described above found an improvement across time in the VABS domains of communication and social abilities but not in daily living skills. Hahn et al. (2015) reported an overall decline in adaptive behavior with age in children with FXS before the age of 10; however, since the latter did not include older participants, it did not solve the question whether these declines plateau or increase during adolescence.

Taking these data as a whole, there is an overall decline both in IQ and in adaptive behavior that is caused by slower development in individuals with FXS across ages, that is stronger in males but present also in females. The cognitive-behavioral phenotype of FXS causes a strong impact on the quality of life of people with FXS and their families (Weber et al., 2019), and empirical studies on medication effects on cognitive and behavioral functioning in FXS are still lacking.

Metformin was originally approved by the Food and Drug Administration (FDA) for its effects in lowering blood glucose levels in patients with type 2 diabetes (T2D). It has been shown that it is safe and effective for the treatment of obesity not only in adults but also in children with and without T2D (Klein et al., 2006; Park et al., 2009; Anagnostou et al., 2016; Muzar et al., 2016). Research in animal models of FXS has demonstrated that metformin can be considered a targeted treatment for FXS, as it can improve both cognitive and behavioral phenotypes of FXS in mice and *Drosophila* models (i.e., Gantois et al., 2017; Monyak et al., 2017; Gantois et al., 2018). Specifically one study found that metformin rescues the social deficits and repetitive behavior (e.g., self-grooming in mice) present in FXS mice models (Gantois et al., 2017). In FXS *Drosophila* model, metformin was found to improve circadian rhythm and memory (Monyak et al., 2017).

The first advances in animal models of FXS led clinicians to use metformin as off-label treatment in patients with FXS. For instance, Dy et al. (2018) found consistent improvement on the Aberrant Behavior Checklist -Community (ABC-C), language and social skills in 7 patients with FXS aged between 4 and 60 after metformin treatment. Later these promising results were replicated also in young children by Biag et al. (2019) who reported a general improvement in development, language, and behavior in nine children with FXS between 2 and 7 years of age after being treated clinically with metformin. In the same year, Protic et al. (2019) described two adult individuals with FXS treated with metformin for 1 year, who exhibited improvements in their IQ scores and behavior, therefore suggesting a possible protective effect of metformin in cognitive decline.

The main aim of this work is to better understand the effects of metformin in assessing both cognitive and adaptive changes over time from the baseline to the open-label follow-up. in FXS. To this purpose, we conducted a longitudinal study in which we compared our cohort of individuals with FXS treated with metformin in an open label format (NCT03722290) with previous data extracted from already published works. More specifically, we examined the differences and similarities in the trajectory of cognitive and adaptive functioning between our participants and the ones reported by Hagerman et al. (1989), Hodapp et al. (1990), Wright-Talamante et al. (1996), Fisch et al. (2002), Hall et al. (2008), Klaiman et al. (2014), Frolli et al. (2015), and Hahn et al. (2015).

## Materials and methods

### Participants and procedures

Twenty-six participants (22 males, 4 females) ages 6 to 25 (mean 13.15 ± 5.50) years old with FXS are included in this study. The diagnosis of FXS and a full *FMR1* mutation was molecularly confirmed through the *FMR1* DNA test on all participants as previously described (Tassone et al., 2008; Filipovic-Sadic et al., 2010).

All participants were recruited from three different sites: the MIND institute (*n* = 10), Edmonton (*n* = 13), and Sainte Justine (STJ) Hospital in Montreal (*n* = 3). Table 1 shows demographic statistics for the study sample by study site and combined. Participants from all three sites responded initially to advertisements and through the clinics to participate in a *Double-Blind, Placebo-Controlled Trial of Metformin in Individuals with Fragile X Syndrome* study lasting 4 months (not yet publish) if they met inclusion criteria including being age 6 to 40 years old with a molecular diagnosis of FXS and IQ that was below 79, male or female and without life-threatening systemic illness. After the controlled trial they were offered an open label metformin follow-up study. The patients reported here have been on metformin for at least 1 year and up to 3 years; see Table 1 for mean follow-up times on the open label study. We remained blinded as to who was treated with metformin or placebo during the controlled trial so those treated with metformin had an additional 4 months of treatment to what is reported as the follow-up times on the open label.

**Table 1:** Descriptive statistics (Mean ± SD, [Min, Max], or *N*(%)) of patient characteristics.

	Total	Edmonton	MIND	STJ	Site difference, *p*-value^*^
	(*N* = 26)	(*N* = 13)	(*N* = 10)	(*N* = 3)	
Sex, Male, *N*(%)	22 (84.6%)	10 (76.9%)	9 (90.0%)	3 (100.0%)	0.765
**Leiter-III**					
Baseline age, years	13.15 ± 5.50 [6.18, 25.85]	12.23 ± 5.68 [6.74, 25.85]	15.46 ± 5.18 [6.18, 23.95]	9.42 ± 3.18 [7.52, 13.09]	0.176
Duration of follow-up, years	1.88 ± 0.63 [1.22, 2.99]	1.71 ± 0.57 [1.28, 2.87]	2.25 ± 0.64 [1.22, 2.99]	1.45 ± 0.23 [1.27, 1.70]	0.050
**Vineland** ^**^					
Baseline age, years	13.09 ± 5.61 [6.18, 25.85]	12.24 ± 5.69 [6.74, 25.85]	15.54 ± 5.50 [6.18, 23.96]	9.42 ± 3.18 [7.52, 13.09]	0.197
Duration of follow-up, years	2.09 ± 1.00 [1.05, 4.80]	1.70 ± 0.55 [1.29, 2.87]	2.88 ± 1.19 [1.05, 4.80]	1.45 ± 0.23 [1.27, 1.70]	0.006

^*^p-value was obtained by Fisher’s exact test or ANOVA for group comparison among the three sites. ^**^A male subject from MIND was excluded from analysis due to missing baseline.

### Timeline

We utilized the baseline IQ and Vineland from the controlled trial and then repeated these measures at their respective study sites in the follow-up period. All participants return at least 1 year after starting metformin treatment for their follow-up visit (see exact follow-up time in Table 1). At their follow-up visit, a repeat Vineland and Leiter-III were administered to each participant. All families signed an institutional review board-approved consent form for the controlled trial and for the open label follow-up studies of metformin treatment.

### Neuropsychological assessment

Clinical data included evaluations performed from January 2020 to June 2023. Tests were generally administered during routine clinical visits; however, since evaluations continued during the pandemic, some changes were made to the standard procedures. For instance, both the examiner and participants used personal protective equipment (PPE) for all the time of assessment and some evaluations (i.e., Clinical interviews) were conducted online.

Cognitive function was assessed using Leiter-III (Roid et al., 2013), which is a tool for evaluating nonverbal cognitive (cognitive battery), memory and attention abilities (memory/attention battery), designed to be administered to individuals without language skills from 3 to 75+ years. The cognitive battery provides a non-verbal intelligence quotient (NVIQ; herein labeled IQ). This is a nonverbal test in terms of both administration of items and participant responses and therefore this made it useful to use across sites because of the difference in language use including both English and French. Changes in cognitive functioning between the baseline and the follow-up visit represent the primary outcome of the study.

### Adaptive behavior assessment

Adaptive behavior was assessed using the Vineland Adaptive Behavior Scales Third Edition (VABS -3) (Sparrow et al., 2016). VABS-3 represents, the gold standard instrument for assessing adaptive behavior and is widely used in research. The interview was administered to the parent/caregiver for subjects. The VABS yields three main domain scores: Communication, Socialization and Daily Living Skills (the fourth, Motor Skills domain, is investigated only in children younger than 7 years of age). VABS-3 also provides an overall Adaptive Behavior Composite (ABC) score, which is calculated by summing up the three domain scores. The VABS-3 has been normed for individuals with intellectual disability and ASD. The third edition includes updated item content to streamline similar items and reduce redundancy, to reflect changes in daily living (e.g., technology) and in conceptions of developmental disabilities (e.g., ASD), and to allow for potential cultural differences by using more generalized wording of certain items.

### Statistical analysis

Statistical analyses of data were performed with an open-source R software version 4.2.3. Results were expressed as mean ± standard deviation (SD) or standard error (SE) of mean, and [median (25th percentile, 75th percentile)], for continuous variables and frequency (%) for categorical variables. For group comparisons, Pearson’s Chi-square test was used for categorical variables and Analysis of Variance (ANOVA) was used for continuous variables. For quantitative variables, normality of the data was assessed using the Shapiro–Wilk’s test prior to statistical analysis. Changes in quantitative variables during the duration of the follow-up from the baseline were analyzed using paired t-test or Wilcoxon signed-rank test as appropriate. Covariate-adjusted analysis was performed to determine changes in quantitative variables using linear mixed-effect models with visit (baseline/follow-up) as a fixed-effect factor, subject as a random-effect factor, and age and sex as covariates. The Benjamini–Hochberg’s false discovery rate (FDR) method was applied for multiple testing correction. FDR < 0.05 was considered statistically significant.

## Results

### Cognitive functioning

**Figure 1:**
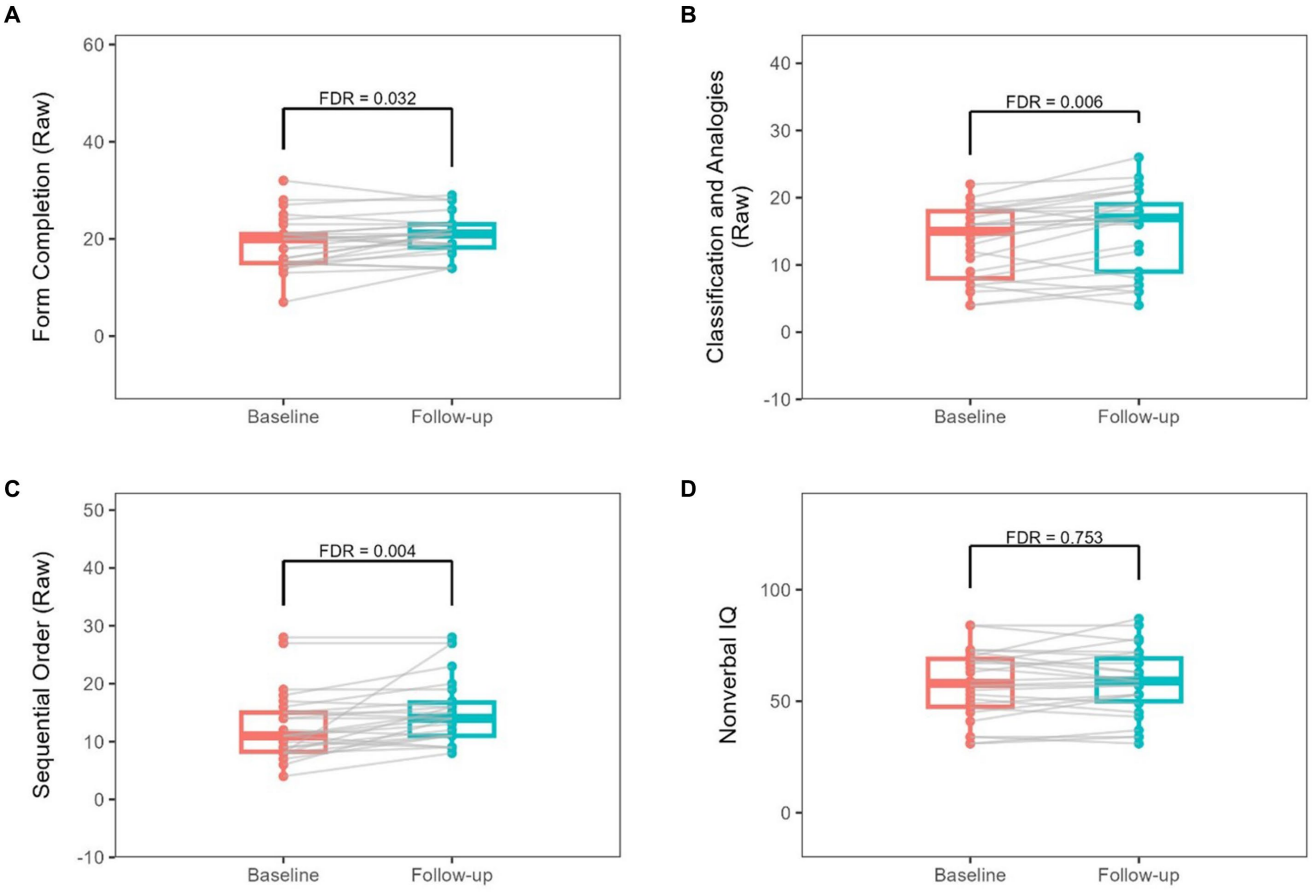
Changes from baseline to follow-up in raw Leiter-III scores. Each gray line represents the longitudinal change for a given participant.

Changes from baseline to follow-up in Leiter-III raw scores are summarized in Table 2. The change over the follow-up duration (1.22–2.99 years, Table 1) is also shown in Figure 1. Form Completion (FC), Sequential Order (SO), and Classification and Analogy (CA) raw scores showed significant increases at FDR < 0.05 (Figure 1; Table 2). Only Classification and Analogy (CA) scaled score showed a significant increase at FDR < 0.05 (Supplementary Table S1). However, none of them remained significant when adjusted for age and sex (Table 3; Supplementary Table S2).

**Table 2:** Changes from baseline to follow-up in raw Leiter-III scores.

Variable	Baseline	Follow-up	Change per year^3^	*p*-value	FDR
Figure ground (Raw)	15.08 ± 4.92	15.31 ± 4.32	0.05 ± 2.24	0.774^1^	0.774
Form completion (Raw)	19.23 ± 5.39	20.65 ± 4.23	0.81 ± 1.79	0.021^1^	0.032
Classification and analogies (Raw)	13.15 ± 5.50	15.24 ± 6.21	0.98 ± 1.54	0.003^1^	0.006
Sequential order (Raw)	12.73 ± 5.81 [11 (8.25, 15)]	15.35 ± 5.67 [14 (11, 16.75)]	1.46 ± 1.99 [1.38 (0, 2.42)]	0.001^2^	0.004
Nonverbal IQ	57.69 ± 15.46	58.35 ± 15.27	0.2 ± 4.32	0.627^1^	0.753

^1^*P*-value was obtained by paired t-test to test significance of change in scores from Baseline to Follow-up. ^2^*p*-value was obtained by Wilcoxon Signed-Rank test to test significance of change in scores from Baseline to Follow-up. For those non-normally distributed variables, summary statistics are reported as Mean ± SD and [Median (Q1, Q3)]. ^3^Change per Year was calculated by the change from Baseline to Follow-up divided by the years of follow-up.

**Table 3:** Covariate-adjusted changes in raw Leiter-III and Vineland-III Scores, controlled for age and sex.

Variable	Estimate of change (SE)	*p*-value^*^	FDR
**Leiter-III**			
Figure ground (Raw)	−0.64 (0.83)	0.447	0.468
Form completion (Raw)	0.45 (0.62)	0.468	0.468
Classification and analogies (Raw)	1.26 (0.65)	0.062	0.155
Sequential order (Raw)	1.73 (0.88)	0.058	0.155
Nonverbal IQ	2.78 (1.62)	0.093	0.155
**Vineland**			
Receptive (Raw)	−0.68 (1.28)	0.600	0.850
Expressive (Raw)	−0.59 (2.35)	0.802	0.869
Written (Raw)	0.48 (1.71)	0.783	0.869
Personal (Raw)	1.93 (2.33)	0.411	0.850
Domestic (Raw)	−2.02 (2.36)	0.399	0.850
Community (Raw)	−2.66 (2.87)	0.361	0.850
Interpersonal relationships (Raw)	−0.2 (2.32)	0.931	0.931
Play, Leisure time (Raw)	2.2 (1.95)	0.264	0.850
Coping skills (Raw)	2.09 (2.03)	0.310	0.850
Adaptive behavior	1.33 (2.32)	0.568	0.850
Communication	−1.13 (2.51)	0.654	0.850
Daily living skills	2.33 (2.83)	0.414	0.850
Socialization	1.75 (2.87)	0.545	0.850

^*^P-value was obtained from a linear mixed effects model to test significance of change in scores from baseline to follow-up, controlled for age and sex.

### Adaptive behavior

**Figure 2:**
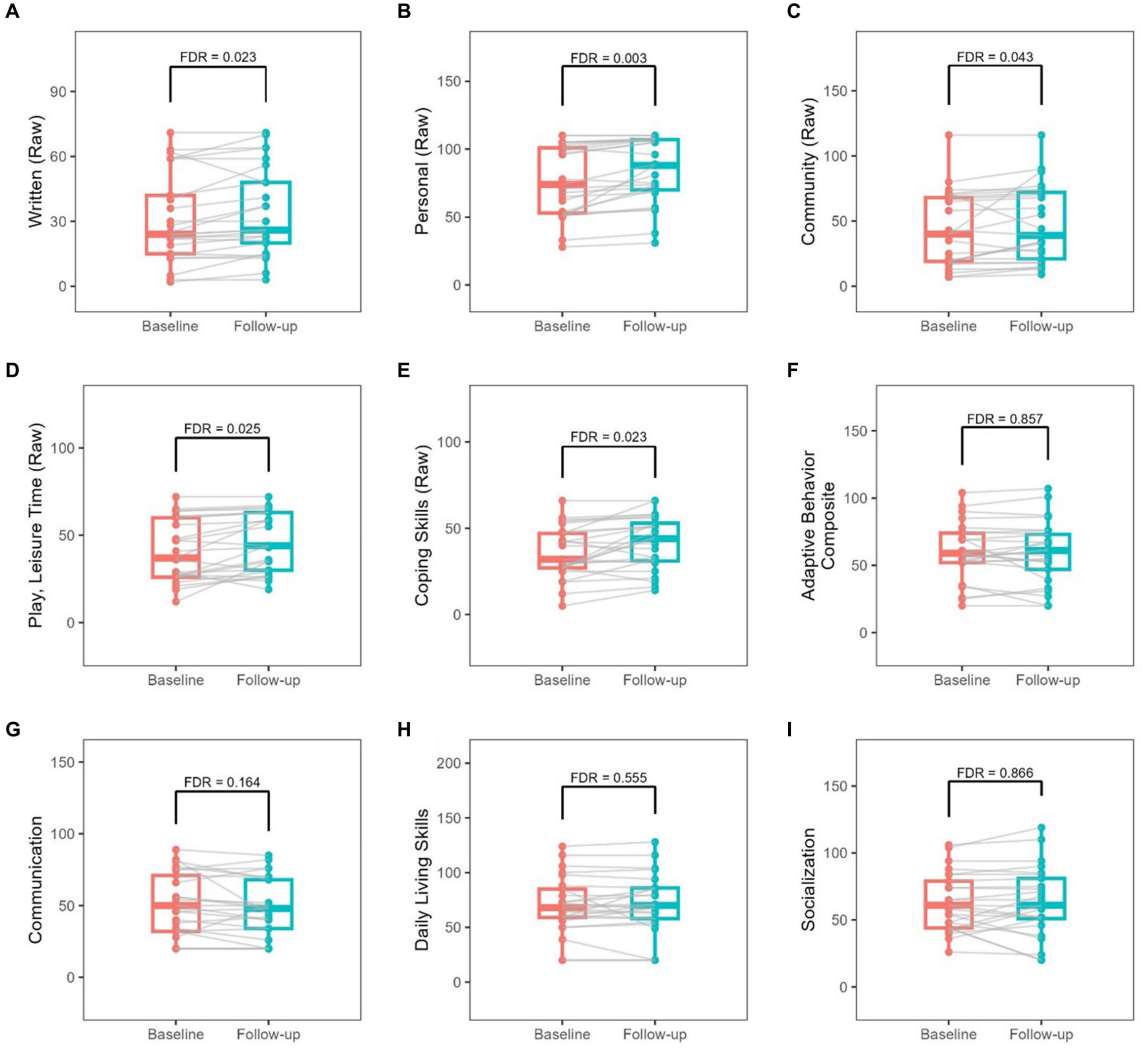
Changes from baseline to follow-up in raw Vineland-III scores. Each gray line represents the longitudinal change for a given participant.

Adaptive behavior score showed no significant change over the duration of the study based on the VABS-3 assessment (Table 4). Change per Year in Vineland raw scores was also calculated and shown in Table 4. Significant increases were found in written, personal, community, play & leisure, and coping skills raw scores at FDR < 0.05 (Table 4; Figure 2). There were no significant changes in scaled scores (Supplementary Table S1). None of scores remained significant when adjusted for age and sex (Table 3; Supplementary Table S2).

**Table 4:** Changes from baseline to follow-up in raw Vineland-III scores.

Variable	Baseline	Follow-up	Change per year^3^	*p*-value	FDR
Receptive (Raw)	58.44 ± 15.02	60.72 ± 15.17	1.12 ± 3.02	0.024^1^	0.056
Expressive (Raw)	75.20 ± 21.00 [81 (57, 96)]	77.76 ± 18.54 [85 (66, 91)]	1.83 ± 4.63 [0 (−0.42, 4.82)]	0.261^2^	0.339
Written (Raw)	30.92 ± 20.59 [24 (15, 42)]	34.16 ± 20.63 [26 (20, 48)]	1.72 ± 2.49 [0.76 (0, 3.12)]	0.004^2^	0.023
Personal (Raw)	76.88 ± 26.02 [74 (53, 101)]	83.88 ± 24.04 [88 (70, 107)]	3.81 ± 4.59 [2.95 (1.04, 4.23)]	<0.001^2^	0.003
Domestic (Raw)	32.84 ± 21.30 [32 (13, 55)]	35.32 ± 20.56 [32 (15, 56)]	1.63 ± 4.68 [0.91 (0, 3.28)]	0.043^2^	0.084
Community (Raw)	42.92 ± 29.13 [40 (19, 68)]	47.60 ± 29.54 [39 (21, 72)]	2.32 ± 6.42 [1.5 (0, 3.4)]	0.016^2^	0.043
Interpersonal relationships (Raw)	56.52 ± 17.54 [55 (41, 69)]	58.80 ± 17.71 [61 (40, 74)]	1.55 ± 4.94 [0.95 (0, 3.66)]	0.077^2^	0.129
Play, Leisure time (Raw)	40.92 ± 17.69	45.52 ± 16.88	2.36 ± 4.1	0.007^1^	0.025
Coping skills (Raw)	36.00 ± 15.17	41.48 ± 15.48	3.08 ± 4.44	0.005^1^	0.023
Adaptive behavior	60.20 ± 21.59 [59 (52, 74)]	59.64 ± 23.23 [61 (47, 73)]	0.24 ± 3.8 [0.68 (−1.59, 2.19)]	0.808^2^	0.857
Communication	50.32 ± 21.92 [50 (32, 71)]	47.40 ± 20.87 [48 (34, 68)]	−0.81 ± 5.47 [−0.61 (−2.78, 0.36)]	0.112^2^	0.164
Daily living skills	70.20 ± 25.92 [68 (59, 85)]	70.72 ± 27.42 [70 (58, 86)]	0.65 ± 4.35 [0 (−1.55, 2.9)]	0.475^2^	0.555
Socialization	63.00 ± 21.78	63.40 ± 25.84	0.72 ± 5.78	0.866^1^	0.866

^1^*P*-value was obtained by paired t-test to test significance of change in scores from Baseline to Follow-up. ^2^*p*-value was obtained by Wilcoxon Signed-Rank test to test significance of change in scores from Baseline to Follow-up. For those non-normally distributed variables, summary statistics are reported as Mean ± SD and [Median (Q1, Q3)]. ^3^Change per Year was calculated by the change from Baseline to Follow-up divided by the years of follow-up.

## Discussion

This initial follow-up study explored the trajectory of cognitive function and adaptive behavior in FXS treated with metformin. The findings thus far indicate that IQ score and adaptive behavior remain stable over the duration of treatment.

The follow-up IQ score among the group (mean age = 13.15 years old) was 58.35 ± 15.27 based on Leiter-III, which is higher than mean IQ score for the 11–15 year-old group based on Stanford Binet (43 ± 14.47) (Dykens et al., 1989). However, different IQ tests were utilized, so it is unclear whether or not there is a significant difference in IQ scores between the treatment group in this study and the non-treatment group discussed in the literature. The stabilization in IQ scores in adolescent years does suggest that the rate of intellectual development did not decline further. We found 3 participants who had approximately a 10-point increase in their NVIQ scores. This has not been documented before in FXS; indeed, most reports in the literature note a decline starting in early adolescent that worsens by the age of 16–20 (Dykens et al., 1989; Hodapp et al., 1990).

Similarly, adaptive behavior in FXS has also been found to decline significantly over time in other studies. The acquisition rate of adaptive behavior slows as FXS individuals age (Klaiman et al., 2014). Significant declines across all the three VABS domains tend to occur in younger age groups compared to older age groups. The study of Klaiman et al. (2014) shows that the change in Vineland ABC score in FXS from age 1–6 was −13.2 (*p* < 0.001) vs. −0.835 from ages 10–14 (*p* < 0.001). In our follow-up study, almost all of the subtest scores on the Vineland (except communication) show improvement and many with statistically significant improvement (see Table 4). However, we were not able to carry out comparative statistics across studies because the standard deviations were not included in the study of Klaiman et al. (2014). Consistent with previous studies, daily living skills was observed to have a mild increase; the change was not significant, likely due to the small sample size. The absence of decline is promising and warrants continued follow-up. The various case reports of improved behavior in FXS with metformin therapy further support the use of this targeted treatment as it has also been shown to have a strong safety profile in both children and adults. In addition to cognition and behavior, metformin targets FXS comorbidities, such as obesity (Biag et al., 2019; Dy et al., 2017). Combined with early decline of adaptive behavior, metformin has more benefits than harm in potentially mitigating cognitive decline and behavior problems in FXS and its comorbidities.

Overall, while there is wide evidence about IQ decline with aging in people with FXS, to our knowledge our work documents stability in cognitive functioning for 1–3 years after treatment with metformin. Indeed, our sample did not exhibit an IQ decrease between the first and the second evaluation. Only a previous work (Protic et al., 2019) described two individuals with FXS who, after being clinically treated with metformin, not only improved in communication and behavior, but also in IQ scores.

Our study has several limitations and the most important is the lack of a control group for this follow-up study. We also have a relatively small sample size, and the follow-up time was limited to greater than 1 year but less than 4 years. An important issue to consider in comparing our sample from previous studies over the last 20 to 30 years is the availability of multiple additional medications and improved therapy programs for those with FXS. For instance, many patients are also treated with an SSRI such as sertraline which has demonstrated benefits in a controlled trial (Greiss Hess et al., 2016) or Cannabidiol (CBD) which has also been shown in a controlled trial to have behavioral benefits (Berry-Kravis, 2022). These medications and others such as a stimulant or guanfacine may work synergistically with metformin to benefit patients. This is also the first study to use the Leiter III in follow-up studies so it does not assess the verbal abilities which may have a different trajectory over time than the non-verbal abilities. The limitation of the IQ tests is another factor that prevents an accurate assessment of FXS individuals. As some participants scored below average in the intellectually disabled range, they could be assigned similar standard scores, which overlooks variations in the floor of the test (Huddleston et al., 2014). In addition, some participants in the double-blind trial may have started metformin therapy during the 4-month trial and the blinding has not been broken yet. However, we made sure that all participants have been on metformin open label for at least 1 year to be included in this follow-up study. Based on these results, it is important to continue following up with participants and obtain cognitive and behavioral assessments at additional ages to understand the trajectory of their development over time. Our goal is to determine whether the IQ and adaptive behavior scores will be stable into adolescence and early adulthood. We will also continue to follow up with eligible participants to increase the power of the study and observe any significant changes in these scores.

## Data availability statement

The original contributions presented in the study are included in the article/Supplementary material, further inquiries can be directed to the corresponding author.

## Ethics statement

The studies involving humans were approved by the University of California Davis School of Medicine. The studies were conducted in accordance with the local legislation and institutional requirements. Written informed consent for participation in this study was provided by the participants’ legal guardians/next of kin.

## Author contributions

PS: Writing – review & editing, Writing – original draft, Investigation, Data curation. FAMM: Writing – review & editing, Writing – original draft, Formal analysis, Data curation. HB: Writing – review & editing, Validation, Project administration, Investigation. MS-A: Writing – review & editing. KK: Writing – review & editing, Validation, Formal analysis, Data curation. MP: Writing – review & editing, Data curation. FT: Writing – review & editing, Methodology, Investigation. AS: Writing – review & editing, Methodology, Investigation. LA: Writing – review & editing. AT: Writing – review & editing. DH: Writing – review & editing, Formal analysis. FB: Writing – review & editing. SJ: Writing – review & editing. SL: Writing – review & editing. RH: Writing – review & editing, Supervision, Resources, Project administration, Methodology, Funding acquisition, Conceptualization.
