# Supplementary material for: Longitudinal follow-up of metformin treatment in Fragile X Syndrome
Source: Front Psychol. 2024 Jun 13;15:1305597. doi: 10.3389/fpsyg.2024.1305597 (PMC11210589; doi:10.3389/fpsyg.2024.1305597)
Supplement: Supplementary file 1 [file Table_1.DOCX]

Supplementary Table S1. Longitudinal changes in scaled Leiter-III and Vineland-III scores.

| Variable | Baseline | Follow-up | Change per Year^3^ | p-value | FDR |
| --- | --- | --- | --- | --- | --- |
| Leiter | | | | | |
| Figure Ground  (Scaled) | 5.19 ± 2.42 | 4.80 ± 2.45 | -0.38 ± 1.46 | 0.233^1^ | 0.435 |
| Form Completion  (Scaled) | 4.31 ± 3.25 | 4.08 ± 2.94 | -0.18 ± 1.1 | 0.301^1^ | 0.435 |
| Classification and  Analogies  (Scaled) | 3.85 ± 3.23 | 5.08 ± 3.39 | 0.55 ± 1.24 | 0.01^1^ | 0.044 |
| Sequential Order  (Scaled) | 4.08 ± 2.06  [4 (3, 5)] | 4.33 ± 1.93  [4 (3, 5.25)] | 0.09 ± 0.85  [0 (0, 0.09)] | 0.784^2^ | 0.784 |
| Vineland | | | | | |
| Receptive  (Scaled) | 8.24 ± 3.55 | 8.56 ± 3.81 | 0.16 ± 1.47 | 0.535^1^ | 0.651 |
| Expressive  (Scaled) | 5.84 ± 5.76  [2 (1, 10)] | 5.12 ± 5.25  [2 (1, 9)] | -0.09 ± 1.62  [0 (-0.78, 0)] | 0.313^2^ | 0.651 |
| Written (Scaled) | 6.40 ± 4.06 | 5.80 ± 4.09 | -0.37 ± 0.9 | 0.096^1^ | 0.365 |
| Personal (Scaled) | 9.08 ± 5.77 | 9.80 ± 5.96 | 0.44 ± 1 | 0.116^1^ | 0.365 |
| Domestic  (Scaled) | 12.32 ± 3.94 | 12.08 ± 5.10 | 0.07 ± 1.45 | 0.727^1^ | 0.727 |
| Community  (Scaled) | 7.92 ± 4.08  [8 (6, 10)] | 7.28 ± 4.17  [7 (6, 9)] | -0.36 ± 1.01  [-0.36 (-0.65, 0)] | 0.054^2^ | 0.365 |
| Interpersonal  Relationships  (Scaled) | 8.60 ± 4.20 | 8.92 ± 4.75 | 0.3 ± 1.5 | 0.527^1^ | 0.651 |
| Play, Leisure  Time  (Scaled) | 7.44 ± 5.08  [7 (1, 12)] | 7.32 ± 5.44  [8 (1, 12)] | -0.14 ± 1.46  [0 (-0.36, 0)] | 0.592^2^ | 0.651 |
| Coping Skills  (Scaled) | 9.92 ± 3.32  [10 (8, 11)] | 10.20 ± 3.57  [10 (8, 12)] | 0.17 ± 1.95  [0 (-0.42, 0.73)] | 0.488^2^ | 0.651 |
| 1. P-value was obtained by paired t-test to test significance of change in scores from Baseline to Follow-up. 2. P-value was obtained by Wilcoxon Signed-Rank test to test significance of change in scores from Baseline to Follow-up. For those non-normally distributed variables, summary statistics are reported as Mean ± SD and [Median (Q1, Q3)]. 3. Change per Year was calculated by the change from Baseline to Follow-up divided by the years of follow-up. | | | | | |

Supplementary Table S2. Covariate-adjusted longitudinal changes in scaled Leiter-III and Vineland-III scores, controlled for age and sex.

| Variable | Estimate of Change  (SE) | p-value* | FDR |
| --- | --- | --- | --- |
| Leiter | | | |
| Figure Ground (Scaled) | -0.26 (0.51) | 0.612 | 0.612 |
| Form Completion (Scaled) | 0.24 (0.4) | 0.557 | 0.612 |
| Classification and Analogies  (Scaled) | 1.29 (0.44) | 0.006 | 0.024 |
| Sequential Order (Scaled) | 0.45 (0.36) | 0.220 | 0.440 |
| Vineland | | | |
| Receptive (Scaled) | 0.01 (0.57) | 0.987 | 0.987 |
| Expressive (Scaled) | -0.65 (0.78) | 0.409 | 0.920 |
| Written (Scaled) | -0.51 (0.45) | 0.269 | 0.807 |
| Personal (Scaled) | 0.67 (0.59) | 0.263 | 0.807 |
| Domestic (Scaled) | -0.1 (0.75) | 0.892 | 0.987 |
| Community (Scaled) | -0.68 (0.48) | 0.170 | 0.807 |
| Interpersonal Relationships  (Scaled) | 0.33 (0.59) | 0.580 | 0.987 |
| Play, Leisure Time (Scaled) | -0.15 (0.61) | 0.812 | 0.987 |
| Coping Skills (Scaled) | 0.26 (0.77) | 0.739 | 0.987 |
| * P-values was obtained from a linear mixed effects model to test significance of change in scores from Baseline to Follow-up, controlled for age and sex. | | | |
